# Targeting the Mechanisms of Resistance to Chemotherapy and Radiotherapy with the Cancer Stem Cell Hypothesis

**DOI:** 10.1155/2011/941876

**Published:** 2010-10-12

**Authors:** Ryan Morrison, Stephen M. Schleicher, Yunguang Sun, Kenneth J. Niermann, Sungjune Kim, Daniel E. Spratt, Christine H. Chung, Bo Lu

**Affiliations:** ^1^Joan C. Edwards School of Medicine, Marshall University, Huntington, WV 25701, USA; ^2^Department of Radiation Oncology, Vanderbilt University School of Medicine, Vanderbilt University Medical Center and Vanderbilt-Ingram Cancer Center Nashville, 1301 22nd Avenue South, B-902 TVC, Nashville, TN 37232-5671, USA; ^3^Department of Hematology/Oncology, Vanderbilt University School of Medicine, Vanderbilt University Medical Center and Vanderbilt-Ingram Cancer Center Nashville, 1301 22nd Avenue South, B-902 TVC, Nashville, TN 37232-5671, USA

## Abstract

Despite advances in treatment, cancer remains the 2nd most common cause of death in the United States. Poor cure rates may result from the ability of cancer to recur and spread after initial therapies have seemingly eliminated detectable signs of disease. A growing body of evidence supports a role for cancer stem cells (CSCs) in tumor regrowth and spread after initial treatment. Thus, targeting CSCs in combination with traditional induction therapies may improve treatment outcomes and survival rates. Unfortunately, CSCs tend to be resistant to chemo- and radiation therapy, and a better understanding of the mechanisms underlying CSC resistance to treatment is necessary. This paper provides an update on evidence that supports a fundamental role for CSCs in cancer progression, summarizes potential mechanisms of CSC resistance to treatment, and discusses classes of drugs currently in preclinical or clinical testing that show promise at targeting CSCs.

## 1. Introduction

Individualized cancer treatment has been an attractive concept since the beginning of cancer research. Breakthroughs in research have allowed the characterization of malignancies according to their unique gene expression, which has allowed the pragmatic targeting of many cancer types based on their specific gene expression patterns. For example, trastuzumab improves the overall and progression-free survival in human epidermal receptor 2- (Her2-) positive breast cancer [1–3]. The receptor-specific monoclonal antibodies bevacizumab [4, 5] and cetuximab [6] have shown remarkable outcome in vascular growth factor receptor- (VEGF-) positive and epidermal growth factor receptor (EGFR)-positive cancer, respectively. Examples of other targeted therapies [7–9] are shown in Table 1. Indeed, the age of individualized cancer therapy has begun.

Individualized profiling and targeting systems provide novel tools to improve both prognostic accuracy and individualized treatment for patients [10]. In addition, each of the classical pillars of cancer therapy—(1) surgical resection, (2) chemotherapy, and (3) radiation therapy—has made significant technological strides, and numerous clinical studies have improved our ability to effectively apply and combine these modalities. Vaccines that prevent the spread of the human papilloma virus (HPV) promise to dramatically reduce cervical cancer; conversely, viruses are being developed that directly attack carcinogenic cells [11]. Advancements such as functional imaging, magnetic resonance imaging, immunohistochemistry, and flow cytometry have recently refined our ability to tease out, subdivide, prognosticate, and define the myriad of permutations in this uniquely complex and malignant disease. A growing arsenal of prevention strategies and screening technologies has allowed physicians to diagnose and treat cancer earlier in its progression than ever before. All of these evolving modalities and strategies to manage cancer have helped result in a pattern of continuously dwindling cancer-related morbidity and mortality in the United States [12].

However, despite this progress, cancer remains the 2nd most common cause of death in the United States [13]. Our continuing inability to cure cancer is largely attributed to the ability of cancer cells to spread and repopulate after initial therapies have eliminated all detectable signs of disease. New interventions that reduce this capacity could have a far-reaching impact on our ability to prevent recurrences, extend survival, and cure many types of cancer. Thus, better understanding the mechanisms of cancer progression for the development of antirepopulation therapies is likely to offer significant clinical benefit. The cancer stem cell (CSC) hypothesis has emerged within this line of investigation. This hypothesis has helped explain how cancer might recur and metastasize despite effective initial treatment and thus represents a promising new front in the war on cancer. 

## 2. The CSC Hypothesis

A paradigm shift in our understanding of cancer tumorigenesis emerged in 1994 when John Dick and colleagues demonstrated that human acute myeloid leukemia (AML) has a hierarchical organization that originates from a primitive hematopoietic cell [14]. This popularized a concept first proposed over a century ago: that cancer growth within a given neoplastic process may be dependent upon only a small fraction of progenitor cells [15, 16]. These cancer cells that retain their normal stem cell properties of self-renewal and pluripotency are often referred to as CSCs. Within the framework of the CSC hypothesis, normally dormant stem cells may inadvertently acquire tumorigenic DNA mutations and become CSCs which inappropriately begin dividing and direct the neoplastic process. Multiple studies have recently provided compelling support of the CSC hypothesis [17–19].

**Figure 1:**
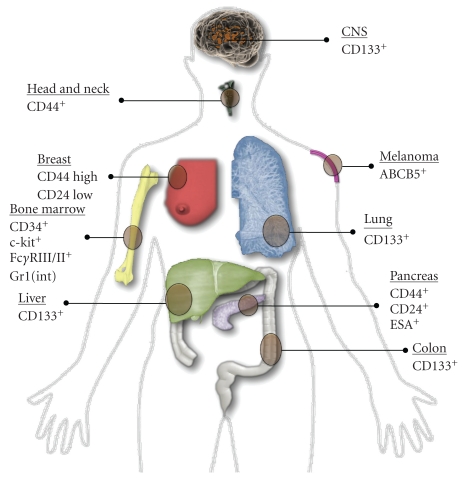
Cell surface phenotype of cancer stem cells. A summary of cancer stem cells surface markers identified in a variety of cancer types.

Despite controversies surrounding the CSC hypothesis [20, 21], substantial evidence has emerged that supports its role in cancer including AML [22], brain [23], breast [24], colon [25], head and neck [26], lung [27], liver [28], melanoma [29], pancreatic [30], prostate [31], and squamous cell [32] cancer (Figure 1). At least 15 drugs designed to exploit the CSC hypothesis have entered clinical trials [33]. However, the CSC hypothesis has not been fully established and will likely evolve as unknown molecular targets capable of promoting tumorigenesis continue to be discovered [34]. Moreover, the translation from the theoretical benefit of CSC eradication into its actual clinical benefit has to be experimentally demonstrated. Another poorly understood nuance is that certain cancer types may be relatively independent of fractional CSC populations, operating more consistently with classical stochastic or clonal evolution models [35]. Although there are limitations to the CSC hypothesis, it is evident that cancer often possesses functionally defined CSCs, and is likely to be at least partially dependent on CSCs for growth and survival. 

In cancer types where neoplastic growth and differentiation depend on CSCs, complete eradication of this population may be curative. Furthermore, agents that force CSCs to rapidly differentiate *en masse* within such cancer types may limit disease progression. Alternatively, suppressing residual CSCs after initial tumor debulking may sustain remissions and extend the progression-free survival of patients receiving CSC suppressive therapy. Considering these distinct therapeutic potentials of targeting CSCs, it appears that CSC-targeted therapies could be an effective complement to traditional treatment approaches such as surgery, chemotherapy, and radiation therapy. Indeed, it is possible that these traditional strategies leave behind residual CSCs which are capable of spreading and regenerating tumors, leading to cancer recurrence and metastasis. Moreover, these recurring tumors often acquire resistance to chemotherapy and radiation [36, 37]. Multiple investigators have demonstrated the ability of CSCs to develop resistance traits after induction chemo- and radiation therapy. 

Evidence suggests that CSCs are highly heterogeneous [38, 39]. This heterogeneity may be responsible for the evolution of resistance to first-line therapies in recurrent cancer since treatment-resistant cells within a heterogeneous tumor population may be selected for during induction therapy. The outcome may be a more aggressive and treatment-resistant malignant recurrence [36]. In addition, CSC heterogeneity may make the pharmacological eradication of the entire CSC population difficult since these cells may exhibit variable expression of drug-targeted genetic markers. This task is complicated by the possibility that cancer may exhibit fluctuating phenotypes, frequencies, and biological properties within an individual patient [40]. Furthermore, existing microenvironmental signaling pathways may recruit or promote CSC functions, perhaps through neoplastic clonal dedifferentiation processes [40, 41]. Until these challenges are overcome, CSC-targeting therapies will not reach their full potential. Regardless, research surrounding the CSC hypothesis has already helped generate numerous potential pharmacological interventions, and combinations of these CSC-specific therapeutic approaches with traditional cancer treatment strategies may show synergistic benefits since their mechanisms of action are distinct and complementary.

## 3. Frequent Cancer Recurrence May Be due to the Preferential Killing of Differentiated Cells While Leaving CSCs behind

As previously mentioned, cancer recurrence may be partly due to the fact that conventional therapies such as chemo- and radiation therapy fail to specifically target CSCs. Instead, these therapies likely enrich CSC populations by preferentially killing differentiated cancer cells that had little potential to sustain cancer growth. Numerous studies indicate that CSCs are resistant to chemo- and radiotherapy and are therefore preferentially preserved when cancer cells are targeted by these approaches [15, 42–45]. Interestingly, during differentiation therapy for the treatment of acute promyelocytic leukemia (APML), all-transretinoic acid and arsenic trioxide are used to induce the differentiation of CSCs down their hematopoietic lineage. The outcome is dramatically reduced self-renewal capacity and extended patient survival [46]. The dramatic anticancer effects of combined modality differentiation therapy in leukemia also demonstrate how synergy between independent therapeutic approaches can achieve remarkable outcomes in cancer therapy [47]. Thus, differentiation treatment of APML serves to illustrate (1) the relative impotence of differentiated cells in cancer, (2) the potential therapeutic benefit of specifically targeting CSCs, and (3) the potential synergy between CSC-specific therapies and existing modalities.

## 4. The Detection and Identification of CSCs

In recent years, an effort has been made to successfully identify stem cells in multiple human malignancies, including hematological, breast, colorectal, brain, pancreatic, and maxillofacial cancer [22, 25, 32, 34, 36, 48–50]. Much attention has been directed to specific cell-surface proteins. Among these, CD133/prominin-1 is a cell-surface molecule thought to be a stem cell marker for multiple cancer types, including CNS, colon, hepatocellular, pancreatic, prostate, and renal cancer [51]. Eramo et al. demonstrated that freshly excised small cell and nonsmall cell lung cancers tissues contain a small subset of CD133-positive cells capable of generating long-term lung tumor spheres *in vitro* and differentiating into tumors *in vivo*. Matsumoto et al. elucidated a mechanistic relationship between CD133 and the hypoxia-inducible factor-1*α* (HIF-1*α*), a downstream molecule in the mammalian target of rapamycin (mTOR) cell signaling pathway, suggesting a role for mTOR in the regulation of CD133 expression [52]. 

In addition to cell-surface markers, many investigators have focused on the selective overexpression of certain genes normally present in progenitor cells. Leukemia cells, which have been transformed from the normally present “partially committed” cells responsible for physiological cellular maintenance, undergo mutations that result in self-perpetuated renewal capabilities. These cells can be identified by selective gene overexpression [24, 53–57]. In an attempt to link cellular pathways to gene expression patterns in lung CSCs, Stevenson et al. compiled and tested a model of 100 signature genes to determine embryonic stemness. Cells with a high embryonic stemness score were found to affect multiple cellular processes, including Ras, Myc, chromosomal instability, and cellular invasiveness [58]. Seo and colleagues demonstrated increased expression of 13 genes in side-population (SP) A549 nonsmall cell lung cancer cells, as compared to non-SP cells [59]. 

Interestingly, Glinsky has developed a novel clinical model to assess the relative “stemness” of cancer cells by quantifying gene expression signatures, and he has shown that this model may predict therapeutic outcomes. The “BMI1 pathway” algorithm is based on a collective signature of 9 individual gene characteristics: TEZ, EED pathway, Suz12/POLII, Suz12, Nanog/Sox2/Oct4, PcG-TF, BCD-TF, ESC pattern, and BMI1 pathway. This multifactorial model allowed the stratification of patients into high-risk and low-risk groups in a retrospective analysis of large cohorts of breast, prostate, lung, and ovarian cancer patients [60]. It remains to be confirmed whether an individual cellular marker can accurately identify normal stem cells or CSCs or whether a multifactorial phenotypic model is required.

## 5. Mechanisms of CSC Resistance to Chemotherapy and Radiation

**Figure 2:**
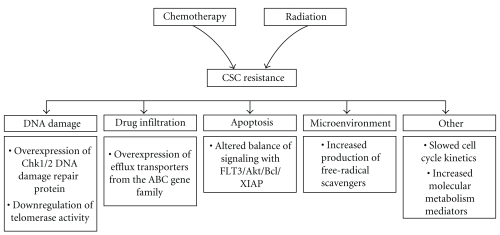
Schematic diagram of the mechanisms leading to cancer stem cell resistance to chemo- and radiation therapy. Cancer stem cells have been found to exhibit a number of genetic and cellular adaptations that confer resistance to classical therapeutic approaches, including relative dormancy/slow cell cycle kinetics, efficient DNA repair, high expression of multidrug-resistance-type membrane transporters, resistance to apoptosis, and protection by a hypoxic niche environment.

CSCs have been found to exhibit a number of genetic and cellular adaptations that confer resistance to classical therapeutic approaches. These include relative dormancy/slow cell cycle kinetics, efficient DNA repair, high expression of multidrug-resistance-type membrane transporters, and resistance to apoptosis (Figure 2). Cancer often acquires resistance to chemo- or radiotherapy after nonlethal exposure [36]. This process likely represents the natural selection of resistant CSCs. Radiotherapy and most types of chemotherapy exert their antineoplastic function by disrupting cancer cell DNA integrity; therefore, it is possible that the oncogenic resistance of CSCs results from increased expression of DNA integrity-maintenance systems [61]. In addition, increased expression of drug efflux pumps may promote oncogenic resistance against cytotoxic chemotherapeutic agents [62, 63].

### 5.1. Resistance to DNA Damage within CSCs

Normal, noncancerous stem cells exhibit well-fortified DNA mutation defense systems that typically serve to prevent mutation into carcinogenic CSCs. Unfortunately, when mutations that create CSCs do occur, the inherent defense systems of stem cells serve to protect them from DNA-targeting chemo- and radiation therapy. The chemo- and radioresistance of CSCs has now been demonstrated in numerous experiments [64], although the mechanisms underlying this resistance are not fully understood. In one experiment, radiation was shown to cause equal levels of damage to all cancer cells, but CSCs were able to repair this damage more rapidly [15]. 

One potential modulator of CSC resistance to DNA-targeting agents is the family of checkpoint kinases 1/2 (Chk1/2 kinases), which become activated after genotoxic stress and arrest the cell cycle to allow DNA repair. These kinases have higher basal and inducible activities in CSCs than in nonstem cells [65]. Supporting the role of Chk1/2 kinases in CSCs, Chk1/2 inhibitors partially reverse the resistance of glioblastoma CSCs to radiation-induced cell death [65, 66]. 

In addition to augmented DNA repair systems, CSCs may also exhibit changes in telomerase function, which allows resistance to chromosomal degradation in these rapidly dividing cells. Telomerase is a complex ribonucleoprotein enzyme that synthesizes and maintains telomeric repeats at the ends of chromosomal strands [67]. Sustained telomerase function is critical in conferring cellular immortality, as telomeres are otherwise shortened with each cell division, eventually triggering cellular senescence. Telomerase function was recently shown to be downregulated in brain CSCs, and several drugs that interfere with telomerase function are already in clinical trials, including arsenic trioxide, GRN163L, and vaccines [68–72]. 

### 5.2. Resistance to Drug Penetration into CSCs

An important component of the DNA integrity defense systems in normal stem cells is the relatively high expression of efflux transporters from the ATP-binding cassette (ABC) gene family [73]. These pumps allow normal stem cells to preserve their genome more effectively against chemical mutagens in an attempt to prevent carcinogenesis. Similar to the way that CSCs may derive resistance to DNA damage from the preexisting DNA repair systems in normal stem cells, CSCs may also derive resistance to chemical mutagens (e.g., chemotherapy) through the expression of drug efflux pumps in normal stem cells from which they were derived. Moreover, the relatively high expression of these transporters may be used to identify CSCs within a neoplasm [74]. Drugs that block the function of efflux transporters or that down-regulate their expression have the potential to overcome CSC chemoresistance. Although multidrug transporters are not likely to significantly influence the direct cytotoxicity of radiation-based therapies, chemotherapy or chemoradiation therapy may benefit from blockade of multidrug efflux pumps in CSCs. 

### 5.3. Resistance to Apoptosis within CSCs

Resistance to therapy might also be conferred to CSCs through the activation of the Akt pathway [75, 76] and the overamplification of apoptosis inhibitor proteins. This was first demonstrated in chemoresistant hepatocellular carcinoma CSCs, which were found to preferentially activate Akt/PKB and bcl-2 cell survival pathways [77]. Moreover, inhibition of Akt by perifosine sensitizes CSCs to radiation-induced apoptosis [78]. This suggests that characterization of Akt and bcl-2 expression in CSCs may have significant clinical utility. FMS-like tyrosine kinase 3 (FLT3) receptor signaling is an important hematopoietic growth pathway upstream of Akt. FLT3 receptors are often mutated in AML and are associated with a high relapse rate and poor prognosis [79]. Inhibition of FLT3 signaling with CEP701 reduces the tumorigenicity of xenografts [80], and CEP 701 has reached phase 2 clinical trials [81].

The mitochondrial pathway of apoptosis is triggered by cytochrome c release and second mitochondria-derived activator of caspase (Smac) activation [82]. Smac, in association with Direct Inhibitor of Apoptosis Binding Protein with low pI (Smac/DIABLO), promotes apoptosis via neutralization of inhibitor of apoptosis (IAP) proteins [82]. Most human cancers have high levels of IAPs, including the X-linked inhibitor of apoptosis protein (XIAP) isoform, which are associated with poor treatment responses [83]. Based on these observations, Vellanki et al. found that the inherent radioresistance of glioblastoma CSCs could be alleviated by promoting apoptosis with an XIAP inhibitor. Importantly, this treatment had no undesirable radiosensitizing effects on normal rat neurons or glial cells [84]. This provides another promising pathway for therapeutic intervention targeting the apoptotic regulation of CSCs.

Another promising molecular target to promote apoptosis in CSCs is nuclear factor *κ*B (NF*κ*B). NF*κ*B is a transcription factor believed to be intricately involved in the development and progression of certain cancer types [85]. Nuclear factor nB (NFnB), a cousin of NF*κ*B, is an antiapoptotic transcription factor that is activated in leukemias [86, 87], pancreatic adenocarcinoma [88], and melanoma [89]. Although these nuclear factors are not as well studied as Akt, they may offer promising drug targets. NF-*κ*B inhibitors include NPI-0052 (salinosporamides A), which is in phase I clinical trials, and TDZD-8 (parthenolide), which is still in preclinical testing.

### 5.4. The Microenvironment and CSCs

Oxygen is a well-known radiosensitizing agent due to its ability to form radiation-induced reactive oxygen species that can damage DNA. Accordingly, radioresistance in breast CSCs may be mediated by increased production of free-radical scavengers [37]. Considering the dependence of radiotherapy on oxygen free radicals, it has long been postulated that areas of low oxygen tension within tumors create microenvironments that are relatively protected from radiation-induced damage [44]. Unexpectedly, it was discovered that CSCs reside along perivascular areas [90] and are thus likely to be well oxygenated [15]. This may help explain the efficacy of antiangiogenic therapies such as bevacizumab in that such therapies may be CSC-specific. Theoretically, CSC compartment hypoxia may be induced by antiangiogenic therapies, conferring radioresistance to the CSCs, although this has yet to be demonstrated *in vivo*, and the clinical significance of this remains unknown. Still, we speculate that radiation might be more effective in treating cancer if it is administered before any antiangiogenic chemotherapies are applied. 

Vermeulen and colleagues recently discovered another interesting role of the microenvironment, specifically in the promotion of cancer cell stemness. They found that high Wnt pathway signaling functionally defines colon CSCs [91]. Importantly, Wnt signaling in these cells depended on costimulation by c-Met signaling. Activated myofibroblasts in the tumor microenvironment were responsible for c-Met activation through production of hepatocyte growth factor (HGF) [91]. Thus, inhibition of stromal-produced HGF or the subsequent activation of c-Met signaling via c-Met inhibitors may represent additional approaches to target CSCs [92].

## 6. Induction Therapy May Enrich CSCs

An important result of the well-documented CSC resistance to radiation and chemotherapy is that these therapies often serve to enrich the resistant CSC subpopulation, perhaps even selecting for more resistant clones within a heterogeneous CSC population. Evidence of radiation-induced enrichment has been shown in both brain [15, 45] and breast [42] CSCs. Furthermore, radiation has little effect on the ability of remaining CSCs to regrow tumors [15]. Thus, CSC enrichment may be the basis for the relative inability of most single modality cancer treatment strategies to control long-term cancer growth. This pattern of initial response followed by long-term failure is known as “the paradox of response and survival in cancer therapeutics” [93]. CSC-specific pharmaceutical interventions are being developed that may eliminate both primary and acquired CSC chemoresistance. This may dramatically improve the treatment of cancer by abrogating the potential for CSC-induced tumor regrowth and systemic disease spread after initial treatment. For example, in experiments by Sung et al. showing that pancreatic CSCs could survive and expand after serial exposures to gemcitabine, this chemoresistance was overcome by the use of CD44 or ABC transporter inhibitors [94]. 

Additional strategies to overcome therapeutic resistance during cancer treatment are as follows.

### 6.1. Concurrent Therapy: The Key to CSC Eradication

It is now well established that combination therapy helps prevent the development of cancer resistance, except in a select group of cancer types where a single pharmaceutically correctable mutation exists [95]. For example, many clinical trials have shown improvements in cancer survival with the use of concurrent chemo- and radiation therapy [96]. This likely reflects the broadly held belief that the best chance for curing cancer is during the first round of therapy before the selection pressure promotes the evolution of resistant CSCs. In later rounds of therapy, not only has the cancer had time to grow and spread further, but it has also evolved resistance to previously encountered therapies. Unfortunately, coadministration of chemotherapy and radiation therapy is not effective against all types of cancer, and it is not always feasible due to its potential significant toxicity. Thus, it will be important to design preclinical studies and clinical trials that evaluate potential synergistic benefits of adding CSC-targeted therapies to traditional cancer regimens.

### 6.2. Surgical Resection Following Induction

As new data supports a role for the CSC hypothesis in solid tumors in addition to hematologic malignancies, outcomes following the surgical resection of solid tumors may significantly improve. If induction approaches can be effectively augmented with anti-CSC therapies, then followup surgical resection may show improved curative outcomes. Theoretically, CSC-specific induction chemotherapies should offer an immediate reduction in CSC metastatic potential and should reduce any hematogenous and lymphatic CSC micrometastases that would otherwise diminish the efficacy of surgical resection. Considering its powerful therapeutic potential, CSC-targeted therapies may be particularly valuable in surgically challenging malignancies such as pancreatic [97] and brain [98] cancer.

### 6.3. Targeting CSCs

In order to more effectively target CSCs, molecular proliferation and survival mechanisms of CSCs must be better understood. Many institutions have developed large banks of malignant tissues with coordinated clinical data, and this resource is being actively mined. Techniques for concentrating, isolating, and enriching CSCs from resected tumors are also rapidly evolving, and cell culture and xenograft models that allow us to transplant and sustain CSCs are maturing. Finally, these advances have been translated into the development of several therapeutic opportunities. Here, we will review some of the prominent classes of drugs that will potentially yield clinical benefits in the near future. An update of clinical trials assessing these targets is illustrated in Table 1 (data from http://clinicaltrials.gov/). 

#### 6.3.1. Wnt Inhibitors

Developmental pathways that direct the differentiation of normal stem cells represent attractive targets for drug discovery. In particular, the roles of Notch and Wnt/*β*-catenin [99] signaling have been examined, and both have been implicated in the development and progression of several types of leukemia [100, 101]. For instance, Wnt signaling serves an important role in promoting the proliferation of immature thymocytes [102]. The nonsteroidal anti-inflammatory drug (NSAID) etodolac inhibits Wnt signaling and may be of benefit in the treatment of chronic lymphocytic leukemia [101]. In fact, all NSAIDS may have anti-Wnt properties and thus potentially have anticancer properties [99].

**Figure 3:**
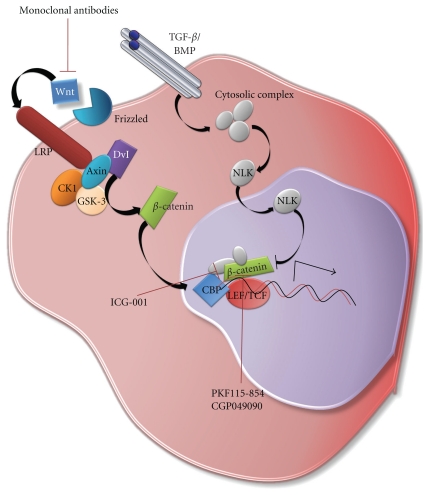
Schematic diagram of the canonical Wnt/*β*-catenin signaling. Wnt/*β*-catenin pathway may promote genomic instability after irradiation, thus allowing tumor cells to both survive after irradiation and develop additional adaptive mutations. ICG-001, PKF115-854, and CGP049040 are anticancer drugs in development that target the Wnt signaling pathway.

The Wnt/*β*-catenin pathway promotes genomic instability and DNA damage tolerance that may be enhanced by DNA damage in CSCs [15]. The pathway has been shown to promote genomic instability in colon cancer [103] and possibly promotes conversion of normal stem cells to CSCs in gliomas [104]. Moreover, it has been shown that high Wnt activity defines colon CSCs [91]. It has been postulated that the Wnt/*β*-catenin pathway may promote genomic instability after irradiation, thus allowing tumor cells to both survive after irradiation and to develop additional adaptive mutations. Wnt inhibitors have been designed to therapeutically prevent this possibility and include ICG-001, fungal derivatives PKF115-854 and CGP049090, as well as monoclonal antibodies against Wnt-1 and Wnt-2 [105] (Figure 3).

#### 6.3.2. Notch Inhibitors

**Figure 4:**
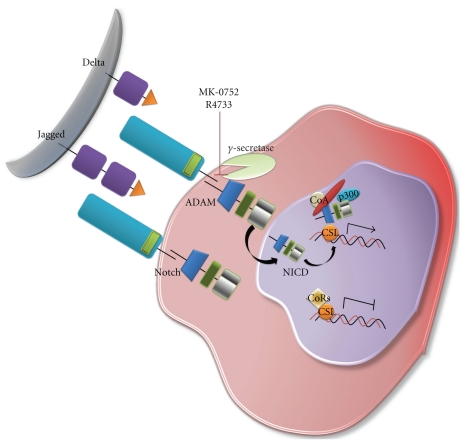
Schematic diagram of the Notch signaling pathway. The Notch/*γ*-secretase/Jagged signaling pathway is an important regulator of differentiation and helps specify cell fate. Notch signaling pathways have been shown to be activated in both breast CSCs and in endothelial cells in response to radiation. MK-0752 and R4733 are drugs under development targeting *γ*-secretase in this signaling pathway.

The Notch/*γ*-secretase/Jagged signaling pathway is an important regulator of differentiation and helps control cell fate [106]. The Notch ligands, Jagged 1 & 2 and Delta1 (D1) to Delta3 (D3), induce the release of the Notch intracellular (Notch-IC) domain via enzymatic proteolytic cleavage by *α*- and *γ*-secretases. Notch-IC translocates to the nucleus where it induces transcription of Notch responsive genes [106, 107]. Notch signaling pathways are activated in both breast CSCs [108] and in endothelial cells [109] in response to radiation. Inhibition of Notch signaling via *γ*-secretase inhibitors can potentially block CSC self-renewal and decrease medulloblastoma growth [110], and significant efforts to downregulate Notch signaling are underway [111]. Currently available Notch signaling inhibitors include MK-0752, a *γ*-secretase inhibitor that is in clinical development for the treatment of leukemia (Figure 4).

In addition to the classical Notch pathway, other routes may be used to modulate the carcinogenic potential of elevated Notch signaling in CSCs. In particular, the Delta/Notch-like epidermal growth factor-related receptor (DNER) can be induced by histone deacetylase inhibition to inhibit the growth of and induce the differentiation of Glioblastoma neurospheres and xenografts [112]. This provides a basis for the manipulation of noncanonical signaling pathways for therapeutic intervention against CSCs.

#### 6.3.3. Hedgehog Inhibitors

**Figure 5:**
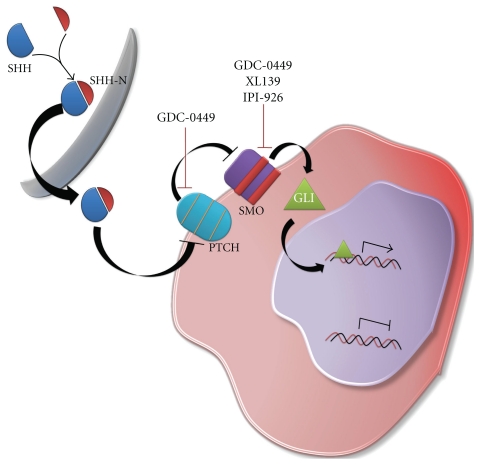
Schematic diagram of the Sonic Hedgehog signaling pathway. The hedgehog signaling pathway is a potential modulator of cancer stem cell carcinogenesis with significant therapeutic implications. GDC-0449, XL-139, and IPI-926 are drugs under development targeting this signaling pathway.

The hedgehog signaling pathway may represent an important modulator of CSC carcinogenesis with significant therapeutic implications [113–117]. Similar to Notch signaling, hedgehog signaling may also benefit from expanded drug discovery efforts within noncanonical pathways [118]. Already, hedgehog inhibitors have been shown to inhibit medulloblastoma growth in mice [119], and at least 3 different hedgehog inhibitors have reached phase I clinical trials (Figure 5).

#### 6.3.4. Targeting the CSC Marker CD133

As mentioned previously, the cell-surface molecule CD133 is believed to be a stem cell marker for multiple cancer types [51]. Its tumor-initiating function has been demonstrated in CNS cancer, where only CD133^+^ cells from brain tumor biopsy samples were able to reform tumors in *in vivo *mouse models [120]. A recent study by Wang et al. demonstrated the potential therapeutic use of targeting CD133 to direct therapy specifically towards CSCs. They conjugated single-walled carbon nanotubules (SWNTs), which allow localized hyperthermia treatment, to anti-CD133 monoclonal antibodies, and cultured these products with both CD133^+^ and CD133^−^ glioblastoma (GBM) cells. A mixture of CD133^+^ and CD133^−^ cells were then exposed to near-infrared laser light, and the CD133^+^ GBM cells were selectively destroyed. They found *in vivo *benefits of this technique as well in mouse models [121].

## 7. Concluding Remarks

With the advent of multidisciplinary approaches to cancer therapy, significant strides have been made in the treatment of cancer. Now with new discoveries relating to CSCs, we have yet another mechanism of therapeutic arsenal that may prove beneficial in combination with current therapeutic modalities. The basic foundations for CSC-targeted therapy are actively being discovered, and there are already several pharmacologic agents available that are capable of specifically modulating CSC intracellular signaling. Still, much remains unknown about the basic signaling mechanisms of CSCs that confer resistance to treatment, and better methods for the disruption of CSC signaling must be developed to fully integrate the CSC hypothesis into our treatment paradigms. Interestingly, CSCs may not necessarily need to be eradicated to prevent cancer progression if they can be forced to differentiate down their lineage *en masse* as they do in the treatment of APML. It is important for future studies to focus on the discovery of new molecular targets for the development of better pharmaceutical agents to eliminate or differentiate CSCs and that these agents be studied in tandem with traditional cancer therapies.

## Figures and Tables

**Table 1:** Update on clinical trials for CSC molecular targets.

Target	Drug	Cancer	Phase	http://clinicaltrials.gov/	Sponsor
				Identifier	
Wnt	Resveratrol	Colon	I, II	NCT00256334	University of California, Irvine
Notch	MK0752	Breast	I	NCT00106145	Merck
		Pancreatic	I, II	NCT01098344	Cancer Research UK
	RO4929097	Renal cell	II	NCT01141569	University Health Network, Toronto
	PF-03084014	Leukemia	I	NCT00878189	Pfizer
Hedgehog	GDC-0449	Solid tumors	I	NCT00968981	Genentech
		Colorectal	II	NCT00636610	Genentech
	PF-04449913	Hematologic	I	NCT00953758	Pfizer
	BMS-833923	Basal cell	I	NCT00670189	Bristol-Myers Squibb
	LDE225	Medulloblastoma	I	NCT00880308	Novartis
